# No modulation of pupil size and event-related pupil response by transcutaneous auricular vagus nerve stimulation (taVNS)

**DOI:** 10.1038/s41598-019-47961-4

**Published:** 2019-08-07

**Authors:** Marius Keute, Mustafa Demirezen, Alina Graf, Notger G. Mueller, Tino Zaehle

**Affiliations:** 1 Department of Neurology, Otto von Guericke University, Magdeburg, Germany; 2 Neuroprotection Group, German Center for Neurodegenerative Diseases, Magdeburg, Germany; 3 Center for Behavioral Brain Sciences, Magdeburg, Germany

**Keywords:** Neuroscience, Neurology

## Abstract

Transcutaneous auricular vagus nerve stimulation (taVNS) bears therapeutic potential for a wide range of medical conditions. However, previous studies have found substantial interindividual variability in responsiveness to taVNS, and no reliable predictive biomarker for stimulation success has been developed so far. In this study, we investigate pupil size and event-related pupil response as candidate biomarkers. Both measures have a direct physiological link to the activity of the locus coeruleus (LC), a brainstem structure and the main source of norepinephrine in the brain. LC activation is considered one of the key mechanisms of action of taVNS, therefore, we expected a clear increase of the pupillary measures under taVNS compared to sham (placebo) stimulation, such that it could serve as a prospective predictor for individual clinical and physiological taVNS effects in future studies. We studied resting pupil size and pupillary responses to target stimuli in an auditory oddball task in 33 healthy young volunteers. We observed stronger pupil responses to target than to standard stimuli. However, and contrary to our hypothesis, neither pupil size nor the event-related pupil response nor behavioral performance were modulated by taVNS. We discuss potential explanations for this negative finding and its implications for future clinical investigation and development of taVNS.

## Introduction

Transcutaneous auricular vagus nerve stimulation (taVNS) is a non-invasive electrical brain stimulation method that has been introduced as an alternative to direct or invasive vagus nerve stimulation (iVNS)^1^. TaVNS is administered to the outer ear, which is partly innervated by the vagus nerve^2^. Alternatively, stimulation can be administered externally to the neck, where the vagus nerve runs next to the carotid artery (transcutaneous cervical vagus nerve stimulation, tcVNS)^3^.

Both iVNS and taVNS/tcVNS can be employed as an adjunct therapy for pharmacoresistant epilepsy^4–6^ and depression^7,8^. TaVNS and tcVNS have been attracting attention in recent years as potential treatments for a variety of further conditions, including chronic headache^9,10^, tinnitus^11^, post-operative cognitive dysfunction^12^, cerebral ischemia^13^, and Alzheimer’s disease^14^. Moreover, several recent studies found effects of taVNS on cognitive and behavioral parameters, including response inhibition^15,16^, executive control of action^17^, and memory^18^. These findings could pave the way for a prospective role of taVNS in neuropsychiatric and neuropsychological therapies.

As of now, the mechanism of action of vagus nerve stimulation is not fully understood, but accumulating evidence suggests that the locus coeruleus – norepinephrine (LC-NE) system is involved: Anatomically, the LC is a downstream projection area of the nucleus of the solitary tract, which is in turn one of the major brain projection areas of the vagus nerve^19^. A number of functional magnetic resonance imaging (fMRI) studies in humans consistently found LC activations following taVNS^20–24^. Moreover, increased levels of NE in the cerebrospinal fluid have been found in rodents after long-term iVNS^25–27^. Electrophysiological studies in rodents^28–30^ found immediate (i.e., beginning within a few milliseconds) LC spiking increases in response to iVNS, scaling with stimulation intensity, pulse width, and frequency.

The LC is the main source of NE in the brain. It has a central role in regulating arousal, attention and adaptive behavior^31–33^. According to an influential model of LC-NE function^34^, there are two functionally distinct modes of LC activity: Tonic activity, leading to a global increase in NE transmission, and phasic activity, leading to an upregulation of NE transmission in response to environmental requirements. Tonic LC activity has been linked to explorative, novelty-seeking, aroused and distractible behavior, whereas phasic LC activity promotes task-engagement and exploitative behavior^35,36^.

Next to invasive LC recordings, pupil size is considered the most reliable noninvasive marker of LC-NE activity, given constant luminance^37,38^, with resting or tonic pupil size being indicative of tonic LC activity and pupillary responses to behaviorally relevant stimuli being indicative of phasic LC activity^39,40^. In this study, we combined taVNS with an auditory oddball paradigm whilst continuously measuring pupil size, asking for effects of taVNS on both tonic and phasic LC-NE activity as indexed through tonic pupil size and event-related pupil dilation (ERPD), respectively.

Our main interest in this study is to improve our mechanistic understanding of taVNS by establishing direct evidence for an effect of taVNS on LC-NE activity in humans. Furthermore, we are interested in pupil size as a candidate predictive biomarker for taVNS efficacy: Previous clinical studies of invasive and transcutaneous VNS in epilepsy and depression patients found that between one third and two thirds of patients did not respond to the stimulation, i.e., showed no amelioration of symptoms^4,41–43^. In order to exploit taVNS to its full potential, it will be necessary to predict individual treatment efficacy and to optimally adapt stimulation parameters. Tonic pupil size and/or ERPD might be used prospectively as an easy-to-use and inexpensive biomarker to identify responders to taVNS and to optimally tune stimulation parameters, given that a clear effect of taVNS on at least one pupillary parameter can be established.

## Results

### Resting measurements

**Figure 1:**
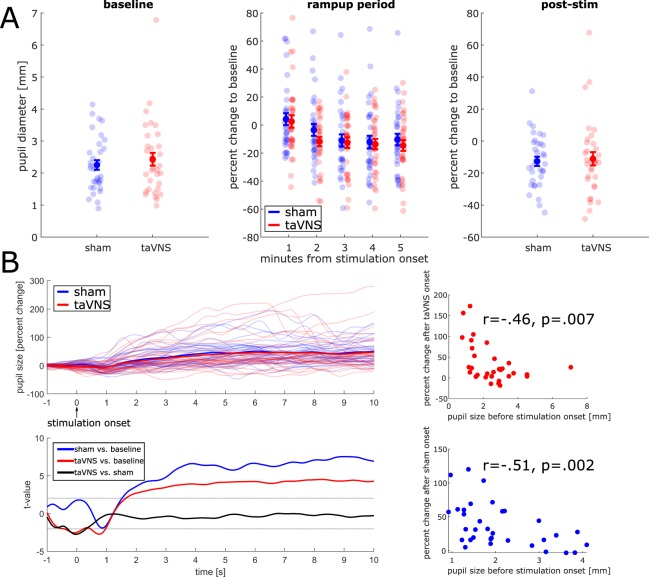
(**A**) Pupil diameter during the baseline measurement (left), change to baseline during the first five minutes of stimulation, and in the post-task resting measurement (~13 min after stimulation offset). (**B**) Left: Grand average pupillary response to stimulation onset and t-values. Dashed lines indicate t = ±2.04, i.e., the (uncorrected) two-tailed threshold for statistical significance at α = 0.05 and df = 32 (33 participants minus one). Right: relationship between mean pupil size in the 2 s before stimulation onset and mean change in pupil size in the first 10 s after stimulation onset. Negative correlations can be seen, consistent with previous studies.

Mean overall pupil size during the baseline period was 2.9 mm (SD 1.3) and was not significantly different between sham and taVNS sessions (*t*_32_ = 0.92, *p* = 0.364, Fig. 1A). Pupil size decreased from the first (before stimulation onset) to the last (~13 min after stimulation offset) resting measurement by 11.9 percent (χ^2^ = 25.2, *p* < 0.001), but was not significantly different between taVNS and sham (χ^2^ = 1.3, *p* = 0.254), nor did the decrease over time interact with stimulation (χ^2^ = 0.1, *p* = 0.738, Fig. 1A). Accordingly, model comparisons based on information criteria (positive values: supporting non-inclusion, negative values: supporting inclusion) favored the non-inclusion of stimulation main effect (ΔAIC = 0.7, ΔBIC = 3.6) and time-stimulation interaction (ΔAIC = 1.9, ΔBIC = 4.8) to the model.

During the rampup period (i.e., during the first five minutes on stimulation), normalized pupil size decreased by 3.7 percent points per minute (χ^2^ = 45.6, *p* < 0.001, Fig. 1A), but was neither different between sham and taVNS (χ^2^ = 0.7, *p* = 0.397, Fig. 1A), nor did stimulation interact with time (χ^2^ = 0, *p* = 0.892, Fig. 1A). Accordingly, model comparisons based on information criteria favored the non-inclusion of stimulation main effect (ΔAIC = 1.28, ΔBIC = 5.0) and time-stimulation interaction (ΔAIC = 2.0, ΔBIC = 5.8) to the model.

Considering the first seconds after stimulation onset, we found an initial increase in pupil size in response to both sham stimulation and taVNS (Fig. 1B, upper panel) compared to a 2 s baseline prior to stimulation onset. It can be seen that the increase lies above the (uncorrected) significance level both for taVNS and sham stimulation compared to baseline (pre-stimulation), but not for the sham vs. taVNS comparison (Fig. 1B, lower panel). This sensory-mediated increase in pupil size was negatively correlated with absolute pre-stimulation pupil size, in line with previous findings (Fig. 1B, right panels)^40^.

### Auditory oddball task

**Figure 2:**
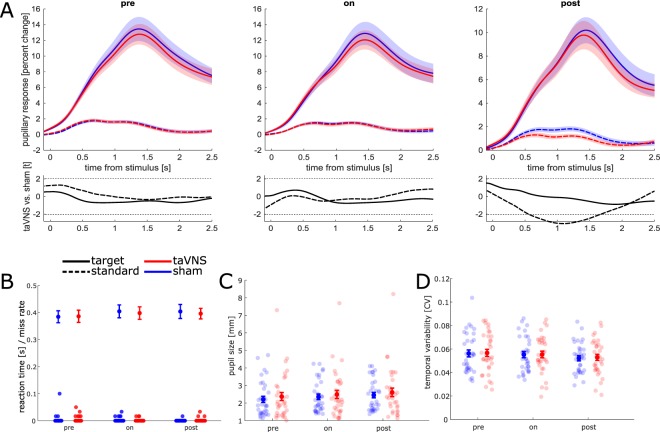
(**A**) Upper panels: Pupillary response to standard and target stimuli in the auditory oddball task before (left), during (middle), and after stimulation (right). Lower panels: t-values comparing sham and taVNS. Dashed lines indicate t = ±2.04, i.e., the (uncorrected) two-tailed threshold for statistical significance at α = 0.05 and df = 32 (Thirty-three participants minus one). (**B**) Mean ± standard error of RT to target stimuli (error bars) and omission error rate (dots). (**C**) Tonic pupil size in the three task runs. (**D**) Temporal variability of tonic pupil size in standard trials, expressed as coefficient of variation (see Methods).

Figure 2A shows the ERPD to target and standard stimuli in the pre-, on-, and post-run. The time- and trial-averaged ERPD was 5.4 percent points higher to target compared to standard stimuli (χ^2^ = 353.5, *p* < 0.001) and decreased by 0.6 percent points per run between the pre-, online-, and post-run (χ^2^ = 17.8, *p* < 0.001), i.e., there was a stronger pupillary response to target stimuli compared to standard stimuli, and this response declined over time. Crucially, there was no significant difference between taVNS and sham (χ^2^ = 0.5, *p* = 0.468), and stimulation did not interact with run (χ^2^ = 0, *p* = 0.939) nor condition (χ^2^ = 0.3, *p* = 0.861), nor was there a three-way interaction between run, stimulation, and condition (χ^2^ = 0.6, *p* = 0.756). Additionally, model comparisons based on information criteria favored the non-inclusion of stimulation main effect (ΔAIC = 1.5, ΔBIC = 5.5), stimulation × run interaction (ΔAIC = 2.0, ΔBIC = 5.9), stimulation × condition interaction (ΔAIC = 2.0, ΔBIC = 5.9), and three-way interaction (ΔAIC = 3.4, ΔBIC = 11.4). Tonic pupil size during the three runs of the oddball task decreased by 0.1 mm per run (χ^2^ = 4.2, *p* = 0.039). There was no significant main effect of stimulation (χ^2^ = 2.6, *p* = 0.108) and, crucially, no run × stimulation interaction (χ^2^ = 0, *p* = 0.977). Model comparisons based on information criteria favored the non-inclusion of run × stimulation interaction (ΔAIC = 2, ΔBIC = 5.8) but were not conclusive on the non-inclusion of stimulation main effect (ΔAIC = −0.6, ΔBIC = 2.7).

Mean overall reaction time (RT) to target stimuli in the auditory oddball task was 0.395 s. RT did not significantly differ between sham and taVNS, nor between task runs, nor did task run interact with stimulation (all χ^2^ < 4.7, all *p* > 0.095). Omission errors to target stimuli were very infrequent (only one subject in one run had an error rate >5 percent, Fig. 2B). No commission errors in response to standard stimuli occurred in any subject.

Mean overall tonic pupil size following standard stimuli (Fig. 2C) was 2.4 mm. It increased by 0.12 mm per run between the pre-, on-, and post-run (χ^2^ = 29.5, *p* < 0.001), but was not significantly different between sham and taVNS (χ^2^ = 0.6, *p* = 0.448), nor was there a run × stimulation interaction (χ^2^ = 0, *p* = 0.938). Model comparisons based on information criteria favored the non-inclusion of stimulation main effect (ΔAIC = 1.9, ΔBIC = 5.2) and run × stimulation interaction (ΔAIC = 1.4, ΔBIC = 4.7).

Mean overall temporal coefficient of variation in standard trials was 0.06, i.e., pupil size varied over time (standard deviation) by 6% relative to the mean pupil size. Temporal variability decreased over the three runs of the auditory oddball task by 0.002 per run (χ^2^ = 15.6, *p* < 0.001, Fig. 2D), but did not differ between sham and taVNS (χ^2^ = 0.1, *p* = 0.773), nor did run interact with stimulation (χ^2^ = 0, *p* = 0.881). Model comparisons based on information criteria favored the non-inclusion of stimulation main effect (ΔAIC = 1.9, ΔBIC = 5.2) and run × stimulation interaction (ΔAIC = 2, ΔBIC = 5.2).

**Figure 3:**
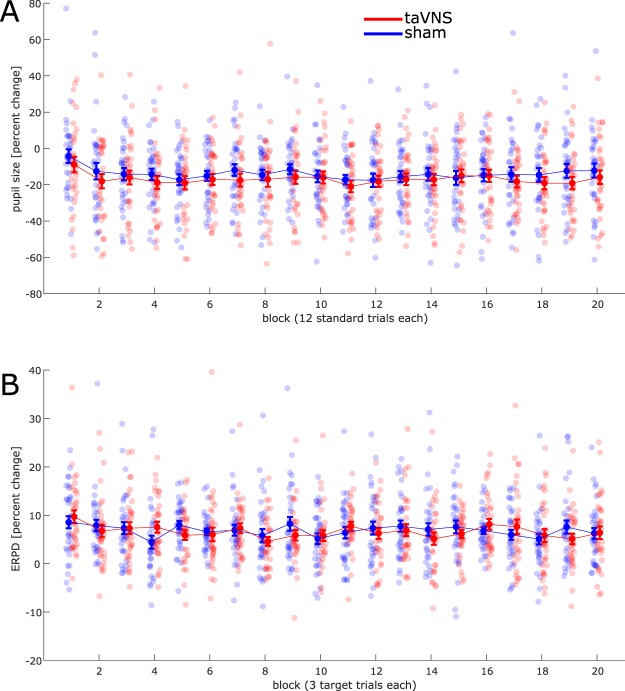
(**A**) Evolution of tonic pupil size over time-on-task during the on-stimulation run of the auditory oddball task, relative to session baseline. (**B**) Time-averaged pupillary responses to target stimuli over time-on-task, relative to pre-stimulus baseline.

Finally, we analyzed the evolution of tonic pupil size and ERPD over time-on-stimulation. To this end, we split standard and target trials from the on-run of the task in 20 blocks, respectively, and computed the mean tonic pupil size from standard trials (normalized to session baseline) and ERPD from target trials (normalized to pre-stimulus baseline). We found that tonic pupil size decreased by 0.1 percent points per block (χ^2^ = 4.8, *p* = 0.029, Fig. 3A), without a main effect of stimulation (χ^2^ = 0.7, *p* = 0.402) nor a block × stimulation interaction (χ^2^ = 0.1, *p* = 0.790). Model comparisons favored the non-inclusion of stimulation main effect (ΔAIC = 1.3, ΔBIC = 6.5) and block × stimulation interaction (ΔAIC = 2.0, ΔBIC = 6.9).

Similarly, we found that ERPD in target trials decreased by 0.05 percent points per block (χ^2^ = 4.0, *p* = 0.047, Fig. 3B), without a main effect of stimulation (χ^2^ = 0.2, *p* = 0.677) nor a block × stimulation interaction (χ^2^ = 0.2, *p* = 0.672). Model comparisons favored the non-inclusion of stimulation main effect (ΔAIC = 1.8, ΔBIC = 7.0) and block × stimulation interaction (ΔAIC = 1.8, ΔBIC = 7.0).

**Figure 4:**
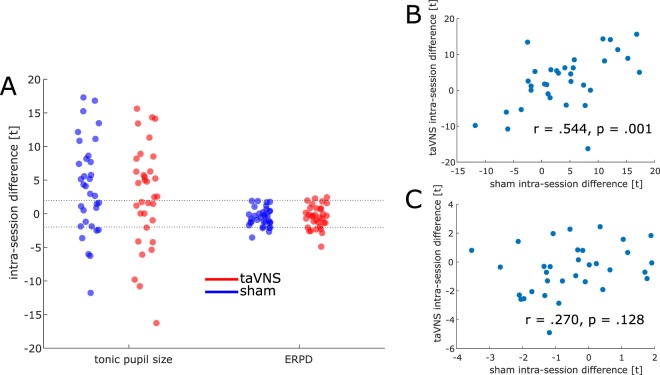
(**A**) Within-session differences between the pre- and on-run of the oddball task for tonic pupil size and ERPD. The dashed lines mark the (uncorrected) two-tailed thresholds for statistical significance at α = 0.05 and df = 239 (tonic pupil size from 240 trials min) and df = 59 (ERPD from 60 trials), corresponding to the number of standard and target trials minus one. (**B**) Relationship between intra-session difference in tonic pupil size in sham and taVNS sessions. (**C**) Relationship between intra-session difference in ERPD in sham and taVNS sessions.

Even though our main focus in this study were overall group-level effects, we carried out additional analyses to capture possible interindividual differences in pupillary stimulation responsiveness. These analyses are summarized in Fig. 4: We computed intra-session differences between the pre- and on-run of the auditory oddball task for tonic pupil size (pupil size over the 240 standard trials) and ERPD (change-to-baseline over the 60 target trials). It can be seen that for ERPD, the intra-session difference exceeded the threshold for uncorrected statistical significance only in a few sessions. For the intra-session change in tonic pupil size, there was considerably greater interindividual variability both in sham and taVNS sessions (Fig. 4A). However, intra-session changes in tonic pupil size were positively correlated between sham and taVNS sessions (*r* = 0.544, *p* = 0.001, Fig. 4B), whereas a clear dissociation could have supported an LC-NE mediated effect (it might have allowed to identify a responder subset of participants, albeit this would still have been rather weak and anecdotal evidence). Conversely, the relatively high correlation suggests that interindividual differences are driven by dispositional factors, such as pupillary responsiveness to somatosensory stimulation or general temporal variability in pupil size rather than by taVNS-induced LC-NE activation. For ERPD, this dissociation can be found (*r* = 0.270, *p* = 0.128, Fig. 4C), but it cannot be interpreted as evidence for an LC-NE-mediated effect, since the overall differences within both taVNS and sham sessions are small and mostly miss statistical significance in within-session comparisons.

## Discussion

We studied the effect of taVNS on tonic pupil size and ERPD. Given that previous studies consistently found LC activation following VNS^23,28^, we had a clear hypothesis that taVNS would increase pupil size. However, our data do not support this hypothesis, in that we did not find any effect of taVNS on neither tonic pupil size nor ERPD.

To the best of our knowledge, there have been four previous studies combining vagus nerve stimulation and pupillometry: One study in rodents^44^ found that resting pupil size of both eyes was increased after unilateral iVNS. Likewise, a human study^45^ found that resting pupil size but not pupillary light reflex was increased under iVNS. This finding could not be replicated by another human study^46^, that found no effect of iVNS on resting pupil size. The only published study of taVNS and pupil size to date^47^ found no effect on resting pupil size in humans (discussed in more detail below). Our study replicates the findings from this study for tonic pupil size in a larger sample, and extends them by also taking into account ERPD.

Our hypothesis was built on previous findings that pupil size is a reliable marker of LC-NE activity^39,40^. Our negative result raises the question of the ‘missing link’ – does it lie in the relationship between taVNS and LC-NE activation, or in the relationship between LC-NE activation and pupil size?

A possible explanation for our negative result could be that stimulation of the afferent auricular vagus nerve fibres through taVNS has no influence on LC-NE activity that is strong enough to entail a measurable pupillary response. In principal, the effect of taVNS on the LC-NE system is well established. Several studies have found acute and sustained effects of iVNS on LC activity and NE concentration^25–28,48^. The number of vagus nerve fibres recruited by taVNS is smaller than for iVNS, because the auricle is innervated only by afferent vagus nerve fibres^2,49^, yet a number of fMRI studies found LC activations following taVNS^21–23^. Our data are in line with a similar, recent study^47^, which did not find effects of taVNS on tonic pupil size nor on the P300 component of the event-related potential in a combined visual and auditory oddball task, which is considered a marker of LC-NE activity, too^40^. However, the same study found that taVNS increased salivary alpha-amylase (sAA), a peripheral marker of central NE level^50^, in line with a previous study^51^.

In sum, there is solid evidence for a modulation of LC-NE activity through taVNS, but it is possible that this modulation does not exceed the threshold necessary to elicit pupil size modulations. This appears to be compatible with previous studies that found effects of iVNS on pupil size^44,45^, given that iVNS can, in principle, reach all types of vagus nerve fibres, whereas taVNS is restricted to afferent fibres^2,49,52^. In this context, it would be interesting so study the effect of tcVNS on pupil size, since tcVNS can engage afferent and efferent vagus nerve fibres, similar to iVNS^52^.

The relationship between LC-NE activity and pupil size, on the other hand, is also well established. Given constant luminance, pupil size, temporal variability of pupil size and ERPD are influenced by a variety of cognitive processes, including attention^53–55^, mental effort^56–58^, emotional arousal^59^, and behavioral relevance of stimuli^60,61^. The mediation of these pupil-behavior relationships through the LC-NE system has been corroborated through electrophysiological LC recordings in monkeys^37,62^ and rodents^63^ as well as pharmacological, behavioral and neuroimaging studies in humans^16,39,40,57,64–66^.

However, pupil size is not exclusively dependent on the LC-NE system. The principal neural mediator of pupil size, next to direct sympathetic innervation of the pupil dilator muscles, is the Edinger-Westphal nucleus, which has no direct anatomical connection with the LC, therefore the relationship between LC activity and pupil size must be mediated by other brain regions, which, in turn, are likely to receive other input next to LC signals^37^. In particular, cholinergic transmission is related to pupil size^63^. Given that vagus nerve stimulation also interacts with cholinergic transmission^67–69^, it is conceivable that interactions between NEergic and cholinergic modulation mask the LC-NE-mediated effect of taVNS on pupil size. However, the interaction between NEergic and cholinergic effects of vagus nerve stimulation is not well understood, and most studies so far have focused on either one, but not both, so we can only speculate about this.

Alternatively, it is also conceivable that we did not find the expected effects because we did not administer proper vagus nerve stimulation. This seems unlikely, however, given that we have demonstrated behavioral and electrophysiological effects of taVNS previously using the same apparatus and similar parameters^16^, and that our results are consistent with a similar, recent study^47^. Moreover, the fact that stimulation onset (taVNS and sham alike) elicited a transient somatosensory pupillary response^70^ (cf. Fig. 1B) and that all subjects reported that they felt the stimulation (taVNS and sham alike) renders it implausible that fundamental technical flaws account for the negative result. Nonetheless, there is an ongoing debate about the optimal location for taVNS^49,71^, which has been kindled by the sparse and not fully consistent knowledge about auricular neuroanatomy. However, a recent neuroimaging study comparing electrical stimulation of tragus, cymba conchae, and other auricular locations found the most consistent NTS and LC activations following cymba conchae stimulation, as applied in our study^22^. Moreover, it is not fully understood how stimulation parameters impact neural effects of taVNS. Only few studies have explored the parameter space in humans^72^, but a recent rodent study applying iVNS^28^ suggests that LC activation is approximately proportional to charge per stimulation pulse, i.e., the product of current intensity and pulse width. The parameters in our stimulation protocol are similar to a previous study combining taVNS and pupil size measurements^47^, but with a shorter total duration of taVNS and a higher current intensity. There is no apparent reason to assume that stimulation parameters account for our negative result. As a potential limitation, it should be noted that the gender distribution in our participant sample is not balanced (27 out of 33 participants male), even though we see no apparent reason to expect gender differences with respect to the questions investigated in our study.

In sum, the most plausible explanation for our negative result appears to be that LC-NE activation through taVNS is not strong enough to affect pupil size/ERPD, since only a subset of vagus nerve fibres are engaged through taVNS.

The apparent absence of an effect of taVNS on both tonic pupil size and ERPD is disappointing, in that pupillometry could have helped the further development of targeted, individualized taVNS administration as an easy-to-use biomarker. However, other candidate biomarkers of taVNS efficacy are under investigation, e.g., spectral power in the M/EEG^73,74^, vagus-sensory evoked potentials^75^, cardiac parameters such as heart-rate variability^76^, and fMRI readouts^43^. As sophisticated, novel taVNS paradigms emerge, such as closed-loop^77^, respiratory-gated^78^, or parameter-optimized^72^ taVNS, the search for such biomarkers will gain relevance, and we think that it should be a focus of future taVNS research.

## Methods

### Participants

Thirty-three healthy young adults (6 female) participated in the experiment. Age range was 21–30 years (M 24.4, SD 1.9). All had normal vision (visual defect of max. ±1 diopter, no glasses or contact lenses could be used with the eyetracking hardware) and were free from any current or past neurological, psychiatric or ophthalmological condition and from any medical or recreational drug intake, except for oral contraceptives (all by self-report).

### Procedure

We carried out a placebo-controlled, single-blind, randomized, within-subjects experimental study. Experimental sessions took place at the German Center for Neurodegenerative Diseases in Magdeburg. Each subject participated in two sessions, one involving sham (placebo) stimulation, and one involving real taVNS. For each subject, both sessions were scheduled in randomized order, at the same daytime and at least 48 hours apart, to enable full wash-out of any stimulation effects. As a reimbursement, subjects received course credit. The study was approved by the ethics committee of the medical faculty at the Otto von Guericke University Magdeburg, and all experimental procedures were carried out in accordance with the Declaration of Helsinki.

Upon arrival, written informed consent was obtained from all participants. They were seated comfortably in a dimly lit room in an adjustable chair, with their head lying on a desk-mounted chinrest. Subjects were instructed to keep their gaze on a black fixation cross presented centrally against a grey background on a 24″ screen at a distance of 70 cm throughout the experiment. Screen luminance had been adjusted in pilot sessions such that gaze could be kept on the screen comfortably over a longer time yet the fixation cross was clearly visible. Eye movements and pupil diameter were recorded continuously from the right eye at a sampling rate of 1000 Hz using a desk-based EyeLink 1000 eyetracker (SR Research, www.sr-research.com).

After an initial baseline measurement of pupil diameter (1 min), subjects performed an auditory oddball task (PRE-run, see below). After the first run, electrical stimulation (taVNS or sham stimulation, see below) started. During the first five minutes of stimulation, subjects had no instruction other than keeping their gaze on the fixation cross (we refer to these first five minutes as ramp-up period). Subsequently, the second run (ON-run) of the oddball task was carried out. After this run, stimulation was turned off and the third run (POST-run) of the task began immediately. The experiment ended with another minute of resting pupil size recording. The experiment was controlled by custom Matlab (Math Works, www.mathworks.com) code using Psychtoolbox 3 (www.psychtoolbox.org) and the Eyelink add-in toolbox for eyetracker control.

### Electrical stimulation

**Figure 5:**
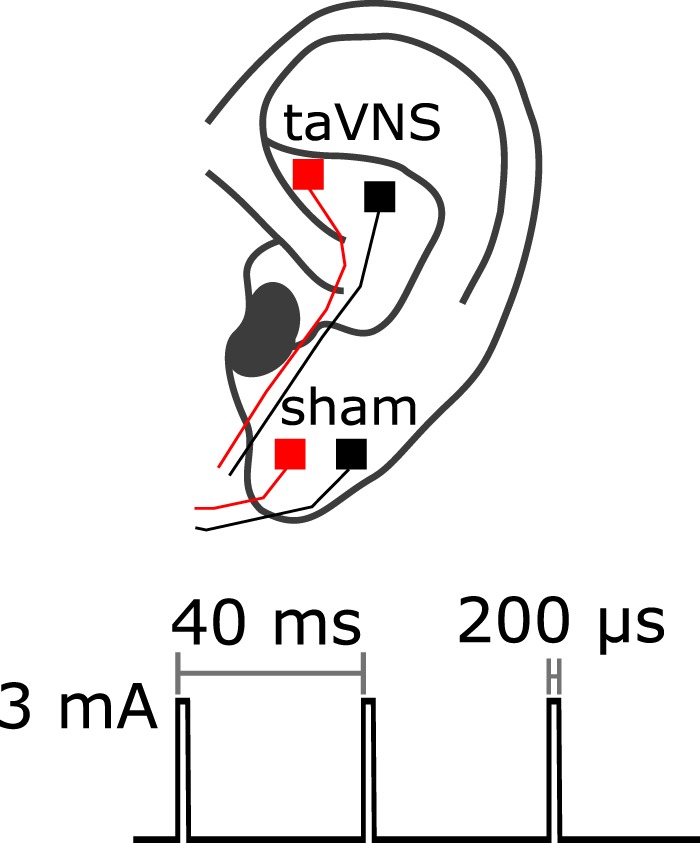
Stimulation setup and current waveform.

TaVNS was administered to the cymba conchae of the left ear, sham stimulation to the left earlobe. Two conventional neurostimulation electrodes were used (Ambu Neuroline, www.ambu.com) that were cut manually to a size of 4 × 4 mm. The two electrodes were mounted 1 cm apart (center-to-center) to a small piece of ear silicone, with the anode being more rostral, and fixated to the skin using Genuine Grass EC2 adhesive electrode cream (Natus Neurology, www.natus.com). Stimulation current was delivered as monophasic square pulses at a pulse width of 200 µs, pulse frequency of 25 Hz and current intensity of 3.0 mA using a medical stimulation device (Digitimer DS7, www.digitimer.com) triggered via a BNC cable by custom code running on an Arduino Uno circuit board (www.arduino.cc). Electrodes were mounted prior to the experiment, and stimulation parameters were tested. All subjects reported that stimulation with the above parameters was perceptible but not painful, both for sham stimulation and taVNS. The stimulation paradigm is illustrated in Fig. 5.

### Auditory oddball task

In each run of the auditory oddball task, 300 auditory stimuli were presented through speakers, comprising 240 standard (500 Hz sinus tones of 60 ms duration) and 60 target (1000 Hz sinus tones of 60 ms duration) stimuli. Standard and target stimuli were presented in pseudo-randomized order, but two target stimuli were always separated by at least three standard stimuli. Inter-stimulus interval (ISI) was randomly jittered between 2.1 and 2.9 s. Subjects were instructed to press the space bar on a PC keyboard with the right index finger after each target stimulus and to ignore the standard stimuli. Total duration of one run was ~13 minutes (with small differences because of the random ISI jitter). The oddball task was designed to resemble a previous task known to reliably elicit ERPD^40^.

### Data analysis

Raw pupil sizes as measured by the eyetracker were linearly transformed from arbitrary units to millimeters^79^. Eyeblinks and other artifacts were identified through a custom-made automatic Matlab procedure, verified by visual inspection and corrected by linear interpolation. On average, 7.7 (±5.8) percent of the data were identified as artifacts and interpolated.

Data from the two baseline measurements (at the beginning and end of the experiment) were averaged over time (1 minute). Data from the ramp-up period (the first five minutes after stimulation onset, without task) were cut to five segments of 1 minute length, and each segment was averaged over time. Data from the three runs of the oddball task were cut to segments from −0.5 to 2.5 s relative to each stimulus. Target stimuli with missed responses were excluded from further analysis. The 0.5 s period preceding each stimulus served as trial-baseline. Event-related pupil dilation (ERPD) was computed as the mean percent change in pupil diameter over 1.5 s post-stimulus relative to the trial-baseline.

To capture the development of tonic pupil size over time-on-stimulation, we additionally computed the mean pupil size over the 2.5 s epochs following the standard stimuli, normalized as percent change to the pre-experiment baseline. Temporal variability of tonic pupil size was computed as coefficient of variation (CV) over the 2.5 s post-stimulus epochs in standard trials, averaged over trials. CV is the standard deviation over time, divided by the mean.

Analyses involving repeated measurements (i.e., pupil diameter or reaction times with multiple trials/measurements per subject) were analyzed using linear mixed-effects regression models. We specified random intercepts and random slopes between sham and taVNS per subject to account for repeated measurements. We used this random effects structure because we found that it fitted the data significantly better than random intercepts only, following recommendations in the literature^80^. Fixed effects were tested by comparing a full model (containing all fixed effects of interest) to reduced models using likelihood ratio tests, leaving out one fixed effect at a time^81^. Models were fit using a maximum likelihood algorithm as implemented in Matlab. Next to the effect size as estimated by the model and the test statistic (likelihood ratio/χ^2^)^82^, we report model comparisons based on Akaike’s (AIC) and Bayes’ information criterion (BIC), two indices of model fit based on model likelihood, penalized by the number of predictors in the model. The difference in AIC or BIC between two nested models (e.g., a model containing a certain fixed effect vs. a model without it) indicates the support for either model through the data^83^. Note that, despite the name ‘Bayes’ in the BIC, these model comparisons do not perform Bayesian inference in the narrower sense, since they are based on penalized likelihood of the data (given the model), but do not incorporate prior and posterior probabilities of the models (given the data).

The datasets and Matlab scripts used for analysis are available from the corresponding author on reasonable request.
